# The Role of Endogenous Lipids in the Emulsifying Properties of Cocoa

**DOI:** 10.3389/fchem.2016.00011

**Published:** 2016-03-11

**Authors:** Joanne M. Gould, Samuel Furse, Bettina Wolf

**Affiliations:** School of Biosciences, The University of NottinghamLoughborough, UK

**Keywords:** oil-in-water emulsions, cocoa, emulsifier, phospholipids, pickering

## Abstract

This paper describes a study in which the emulsifying properties of cocoa material with and without its lipid fraction were explored. This study was motivated by the commercial interest in naturally-occurring particulate emulsifiers as opposed to the chemically modified emulsifying particles presently available for commercial use. The hypothesis was that endogenous lipids from cocoa were responsible for driving the formation of stable oil-in-water (o/w) emulsions. The data presented includes relative quantification of phospholipids from different commercially available cocoa material using ^31^P NMR spectroscopy and analyses of the emulsifying power of delipidified cocoa material. The commercially available cocoa material comprised several phospholipids, with phosphatidylcholine being the most abundant in all samples. Dispersions of delipidified cocoa material were found to drive the formation of o/w emulsions despite the absence of lipids. We therefore concluded that the emulsifying behavior of cocoa material is not entirely reliant upon the endogenous lipids. This suggests that cocoa material may have a new and potentially widespread use in industrial food preparation and may inform manufacturing strategies for novel food grade emulsifiers.

## Introduction

Emulsions comprising oil as the dispersed liquid fraction and water as the continuous one are found across a considerable variety of foods, including soups, salad dressings, mayonnaise, sauces and the majority of dairy products. The emulsification of oil into water to produce such oil-in-water (o/w) emulsion based consumer goods requires the addition of an emulsifying agent. Such agents facilitate emulsion processing through reduction in interfacial tension and contributing to emulsion stability during shelf-life by counteracting thermodynamically driven instability mechanisms. Typical emulsifying agents in foods are amphiphilic proteins and surfactants such as lecithin, although, in recent years, the use of solid particles as emulsifiers has attracted significant research interest (Dickinson, 2012; Lam et al., 2014; Berton-Carabin and Schroën, 2015). Solid particles have been reported to impart a higher emulsion stability with respect to proteins and surfactants, as their energy of desorption from the interface is several orders of magnitude higher than in the case of proteins and surfactants (Aveyard et al., 2003; Hunter et al., 2008). Thus, coalescence and Ostwald ripening are less favored, imparting prolonged microstructure stability which in turn increases the shelf-life of edible emulsion based consumer goods. This property also facilitates formation of stable multiple emulsions, a system that is sought-after for low fat food formulations (Lobato-Calleros et al., 2008; Dickinson, 2011), and for the encapsulation of bioactive species for targeted release (Lamba et al., 2015; McClements, 2015). An additional attraction of formulating food emulsions with particulate material is that artificial surfactants are not required. However, to date, the application has been limited due to the scarcity of food grade particulate emulsifier ingredients.

Recently, we demonstrated that o/w emulsions comprising only sunflower oil, water and a particulate material from several parts of the *Theobroma cacao* bean showed no evidence of emulsion instability in form of droplet coalescence (≥100 days) or free oil (≥2 years) (Gould et al., 2013) for storage periods well above the requirements of most emulsion based manufactured foods. While the use of food grade particles is an obvious requirement for application in the food industry, cocoa material is not only food grade but can be classified as natural emulsifying food particles as there is no requirement for chemical modification to impart emulsifying ability. This is in contrast to hydrophobised starches (Yusoff and Murray, 2011); the only particulate emulsifier ingredient applied in the food industry to date.

The efficacy of cocoa material as an emulsifier (Gould et al., 2013) raises the question of which molecular species is/are responsible for it. It has been understood for some time that cocoa comprises up to around 0.4% (*w/w*) phospholipids (Knapp, 1937; Parsons et al., 1969). Phospholipids, as a class of biomolecules, are well-established emulsifying and surface-active agents in the context of food (Guzey and McClements, 2006; Singh et al., 2009; Pichot et al., 2013). The presence of such species in this quantity is consistent with the commonly used food emulsifier lecithin, which contains a mixture of phospholipids (Furse et al., 2013), and can drive the formation of o/w emulsions at concentrations as low as 0.5% (*w/w*) (Pan et al., 2002). The presence of these species in all of the cocoa material therefore led us to the hypothesis that the cocoa material used previously (Gould et al., 2013) was an emulsifying agent because of the presence of phospholipids.

In order to determine the contribution of such endogenous lipids to the kinetic stability of the microstructure of o/w emulsions with cocoa material, the phospholipid fraction of four different types of cocoa was profiled using ^31^ P NMR. Surface tension and emulsion assays were used to determine the interfacial functionality of both the untreated and the delipidified cocoa material and, by comparison, indicate what contribution non-lipid species make to the emulsifying power of cocoa material.

## Materials and methods

### Cocoa material

Cocoa powders (CPs) were a gift from Barry Callebaut (Banbury, Oxfordshire, UK) and cocoa fiber (CF) was donated by Food Ingredient Technology (Sandy, Bedfordshire, UK). Data including the total lipid content are given in Table 1. The samples codes used in a previous study are included for comparison (Gould et al., 2013).

**Table 1:** **Cocoa material investigated for impact of lipid composition on emulsifying ability**.

**Sample code**	**Sample description**	**Total lipid fraction according to supplier specification [% (*w/w*)]**	**Equivalent sample in (Gould et al., 2013)**
CP-l	Fat-reduced alkalized cocoa powder	< 1	CP1(1)
CP-m	Medium brown alkalized cocoa powder	10–12	CP5(10–12)
CP-h	Medium brown alkalised cocoa powder	20–22	CP10(20–22)
CF	Cocoa fiber	5	CF(5)

*The sample code signifies whether the cocoa material is a cocoa powder (CP) or a cocoa fiber (CF) with the size of the lipid and triglyceride fraction indicated for the CPs as l, low; m, medium; and h, high. The fourth column shows the sample coding used in previous work (Gould et al., 2013)*.

### Lipid extraction

Extraction solvents (petroleum ether, chloroform, ethanol and triethlyamine) were HPLC grade (Sigma-Aldrich, Gillingham, Dorset, UK) and used without further purification. CUBO solvent for NMR was prepared freshly before use following published protocol (Bosco et al., 1997; Culeddu et al., 1998; Cremonini et al., 2004) using guanidinium chloride (Fisher scientific, Loughborough, UK), deuteriated dimethylformamide (*d*_7_-DMF) (Sigma-Aldrich, Gillingham, Dorset, UK) and triethlyamine. NMR tubes were obtained from Wilmad (Vineland, NJ, USA). To extract the triglyceride fraction, a 50 g sample of cocoa was mixed with petroleum ether (300 mL) and agitated (1 h at 4°C). The remaining solid was isolated by centrifugation (11,400 × *g*, 1 h, 4°C; J2-21M Induction Drive Centrifuge, Beckman, High Wycombe, Buckinghamshire, UK). The solid was re-suspended in CET (Furse et al., 2013, 2015a) (chloroform: ethanol: triethylamine, 3:1:1, 300 mL) and agitated (1 h at 4°C) to extract the phospholipid fraction. The organic solutions were concentrated to dryness separately under reduced pressure. The cocoa solids were suspended in CET for a second time, agitated, filtered and the organic solution dried; however no further lipid was extracted. All traces of solvent were removed by drying the delipidified cocoa (40°C, 4 days, Vacuum Oven, Weiss Gallenkamp, Leicester, UK). Three independent extractions were carried out for each type of cocoa material.

### Profiling of lipid fractions

Lipid isolates (80 mg) were dissolved in 200 μL CUBO solvent and after agitation centrifuged at 21,100 × *g* for 30 min at 4°C (Heraeus Fresco 21 Microcentrifuge, Thermo Corporation, Waltham, USA). The organic solution was then transferred to a 5 mm NMR tube and diluted (CUBO solvent, overall sample volume 500 μL). The ^31^P NMR spectra were obtained from a Brucker AV400 spectrometer (Brucker, Coventry, UK). The phospholipids were identified using the reported shifts of phosphorous resonances (Bosco et al., 1997; Culeddu et al., 1998; Cremonini et al., 2004; Furse et al., 2013), which were 5.12 ppm for phosphatidic acid (PA), 1.25 ppm and 1.21 ppm for phosphatidylglycerol (PG), 1.07 ppm for phosphatidylinositol (PI), 0.48 ppm, and 0.44 ppm for *lyso*-phosphatidylcholine (LPC) and phosphatidylcholine (PC) was calibrated to 0.00 ppm. NMR data was processed (including deconvolution) using TopSpin 2.0 and 3.1.

### Emulsion preparation

The oil phase of the emulsions consisted of commercially available sunflower oil (J Sainsbury Plc, London, UK) and double distilled water. Surface active impurities present in sunflower oil were removed by adding magnesium silicate [4% (*w/w*), Sigma-Aldrich, Dorset, UK] followed by stirring (30 min, 600 rpm, RCT Basic, IKA—Werke GmbH & Co, Staufen, Germany). The magnesium silicate was removed by centrifugation (2700 × *g*, 30 min, Jouan CR3i multifunction Centrifuge, Thermo Fisher Scientific, Massachusetts, USA). Absence of surface active molecules in the purified oil was confirmed by measuring interfacial tension against water using the pendant drop technique (see method below) verifying that interfacial tension at 20°C was constant at a value of 27.3 ± 1.5 mN.m^−1^. It remained unchanged for at least 29 days of storage at room temperature in the dark which was the longest storage period for purified oil used for emulsion preparation in this study. Sodium azide (Sigma-Aldrich, Gillingham, Dorset, UK) was added as anti-microbial agent to all aqueous emulsion phases at a final concentration of 0.02% (*w/w*). Emulsions (o/w, 20% (*w/w)* oil) were produced on a 100 g scale. Water (75.2 g) and cocoa material (4.8 g) were placed in a glass beaker (250 mL) and mixed briefly by hand to produce an aqueous dispersion of 6% (*w/w*) cocoa material. Sunflower oil (20 g) was added to the dispersion prior to homogenization using a high shear overhead mixer (L5M Series fitted with emulsor screen, Silverson, Chesham, Hertfordshire, UK) operating at 8000 rpm for 2 min.

### Characterization of emulsions

Particle size analysis was used to assess the coalescence stability of the emulsions; no significant increase in droplet size over the storage period deemed a stable emulsion in this study. Size distributions of prepared emulsions were measured with a low angle laser diffraction particle size analyser (LS 13 320, Beckman Coulter, High Wycombe, UK) fitted with an aqueous dispersion cell (Universal liquid module, LS13 320, Beckman Coulter, High Wycombe, Buckinghamshire, UK). Data was analyzed using the Fraunhofer approximation optical model from the instrument's software. Graphical representations of the surface area based mean, d_3, 2_, are presented. Three independent measurements of each sample were used to calculate the values given.

### Interfacial tension and surface tension measurement

A drop shape tensiometer (PAT-1, Sinterface, Berlin, D) was used to quantify interfacial tension and surface tension of samples. Interfacial tension measurement was used to confirm the absence of surface active impurities in the oil, whereas the surface tension of the aqueous dispersions of cocoa material was used to assess the contribution of the lipid fraction to the surface activity of cocoa. All measurements were taken at 20°C and values recorded for 600 s after drop formation. The values reported in the results section represent an average of the equilibrium surface tension, recorded at 600 s. Three independent measurements of each sample were used. For interfacial tension measurement at the purified sunflower oil/water interface a straight capillary with a diameter of 2 mm (outer diameter) was used to dose a water drop of 35 mm^3^ constant volume into the oil phase contained in a quartz glass cuvette. The surface tension of aqueous dispersions of 6% (*w/w)* cocoa material was measured using the same straight capillary and constant drop volume of 35 mm^3^. Due to the low surface tension of the 6% (*w/w)* aqueous dispersion of cocoa fiber the volume of the droplet was reduced to 25 mm^3^ for this sample. It was previously verified that reducing the droplet volume did not change the result.

### Statistical analysis

Mean droplet size and standard deviation are reported based on three independent samples. Whether or not significant increases in emulsion droplet diameter occurred over storage was determined using an ANOVA and Tukey's statistical test was carried out. In order to compare the mean droplet diameter of emulsions stabilized by the same type of cocoa material either untreated or delipidified, a *t*-Test was used to assess difference between the two samples. The level of significance was set at *p* = 0.05 for both statistical tests.

### Results

The cocoa used in this study reflected the cocoa material from different parts of the cocoa bean, and different treatments during isolation. The latter represent the range of sizes of the lipid fraction in commercially available cocoa and are low, medium and high lipid and denoted CP-l, CP-m, and CP-h, respectively. The fourth cocoa material is powdered cocoa fiber (CF).

### Endogenous lipid composition

**Figure 1:**
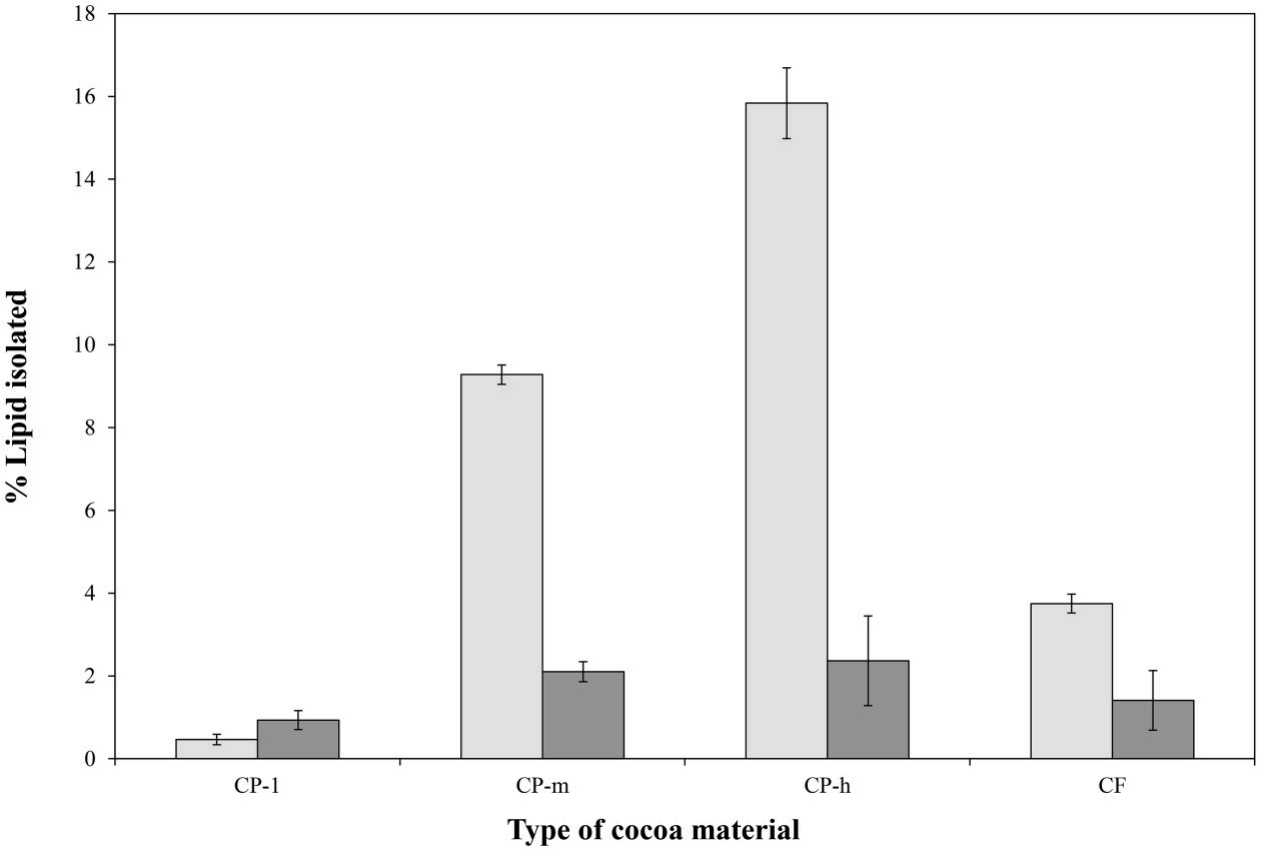
**Relative sizes of the phospholipid and triglyceride fractions of four cocoa materials**. The percentage of lipids and triglycerides isolated was based on initial dry sample weight and the triglyceride (TAG,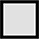) and phospholipid (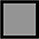) proportions are shown. CP-l, m and h indicate the low, medium, and high lipid and triglyceride fraction of the original cocoa powders, respectively. The error bars represent standard deviation.

**Figure 2:**
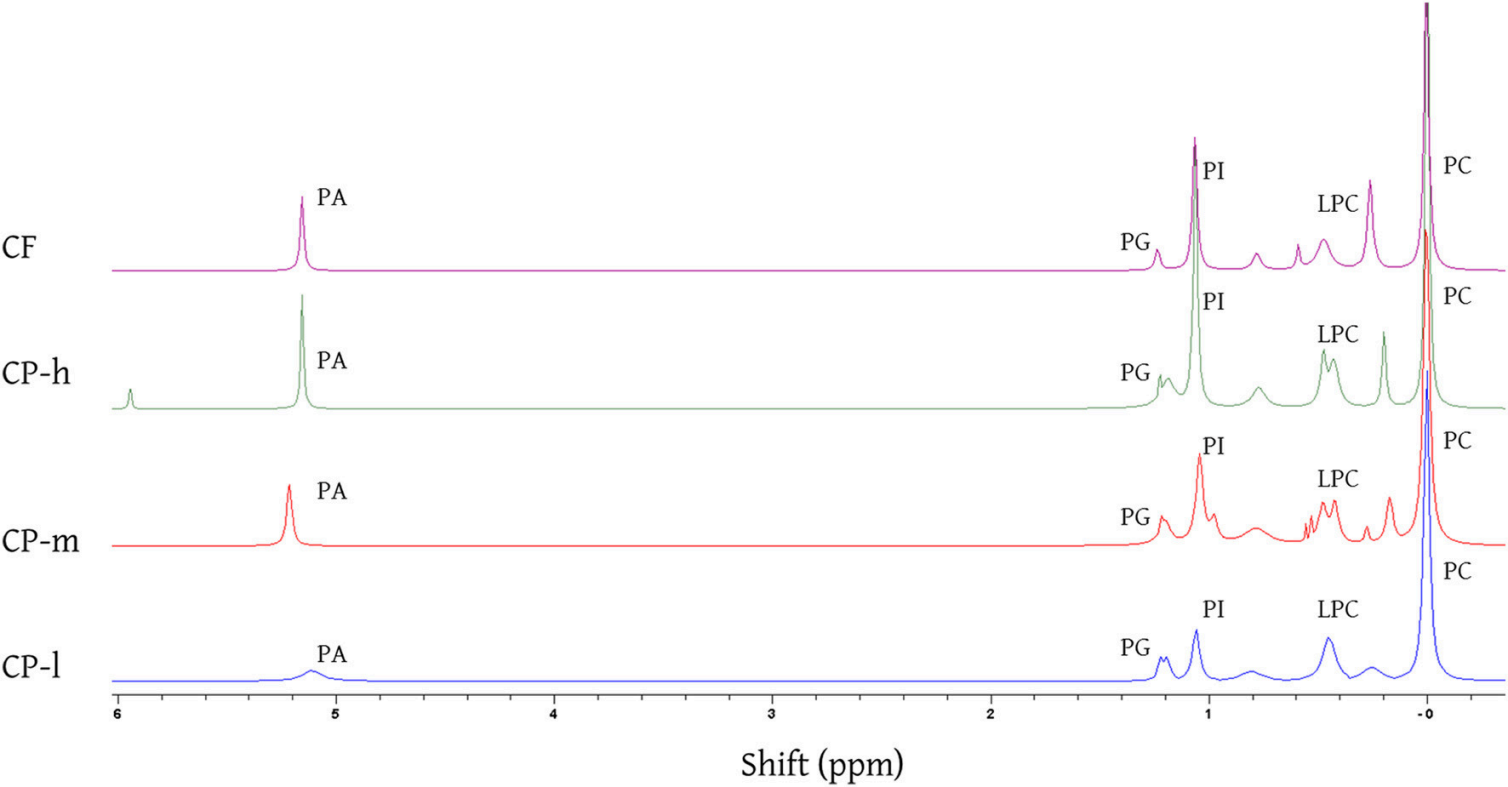
**^31^ P NMR spectra of the phospholipid fraction of the cocoa lipid extracts acquired by CET (chloroform, ethanol, and triethylamine) solvent extraction**. The identity of the phospholipids was established using reported shifts of phosphorous resonances for phosphatidic acid (PA), phosphatidylglycerol (PG), phosphatidylinositol (PI), *lyso*-phosphatidylcholine (LPC), and phosphatidylcholine (PC). Assignments made by assigning the PC resonance to 0·00 ppm and measuring the relative shift of each resonance and comparing with literature values (Cremonini et al., 2004; Furse et al., 2013, 2015b). CP-l, m and h indicate the low, medium and high lipid and triglyceride fraction of the original cocoa powders respectively.

A sequential extraction approach (Furse et al., 2015a) was adopted in order to separate the triglyceride (TG) fraction and the phospholipid (PL) fractions clearly. This approach has been shown to remove more than 99.99% of the TGs and thus gives practically no contamination of the PL fraction (Furse et al., 2013). The triglyceride fraction of the cocoa materials was therefore isolated using petroleum ether, after which the PL fraction was isolated using the CET solvent system (Furse et al., 2013) The size of the TG and PL fractions was assessed gravimetrically (Figure 1) and the profile of the PL fraction determined using^31^P NMR spectroscopy (Figure 2).

The overall size of phospholipid fraction of the cocoa varied between 0.9% (*w/w*) and 2.4% (*w/w*) for the CPs with the lowest (CP-l) and the highest (CP-h) lipid content, respectively.

^31^P NMR spectroscopy showed that the same five phospholipids PA, PG, PI, LPC, and PC are present in all cocoa material, regardless of the mass or profile of the lipid fraction or origin (Figure 2). Signals were identified using literature values for chemical shift (Murgia et al., 2003; Furse et al., 2013, 2015b). Phosphatidyl ethanolamine and phosphatidyl serine have been previously identified in cocoa beans (Parsons et al., 1969) but were not found in all the cocoa material tested in this study.

**Figure 3:**
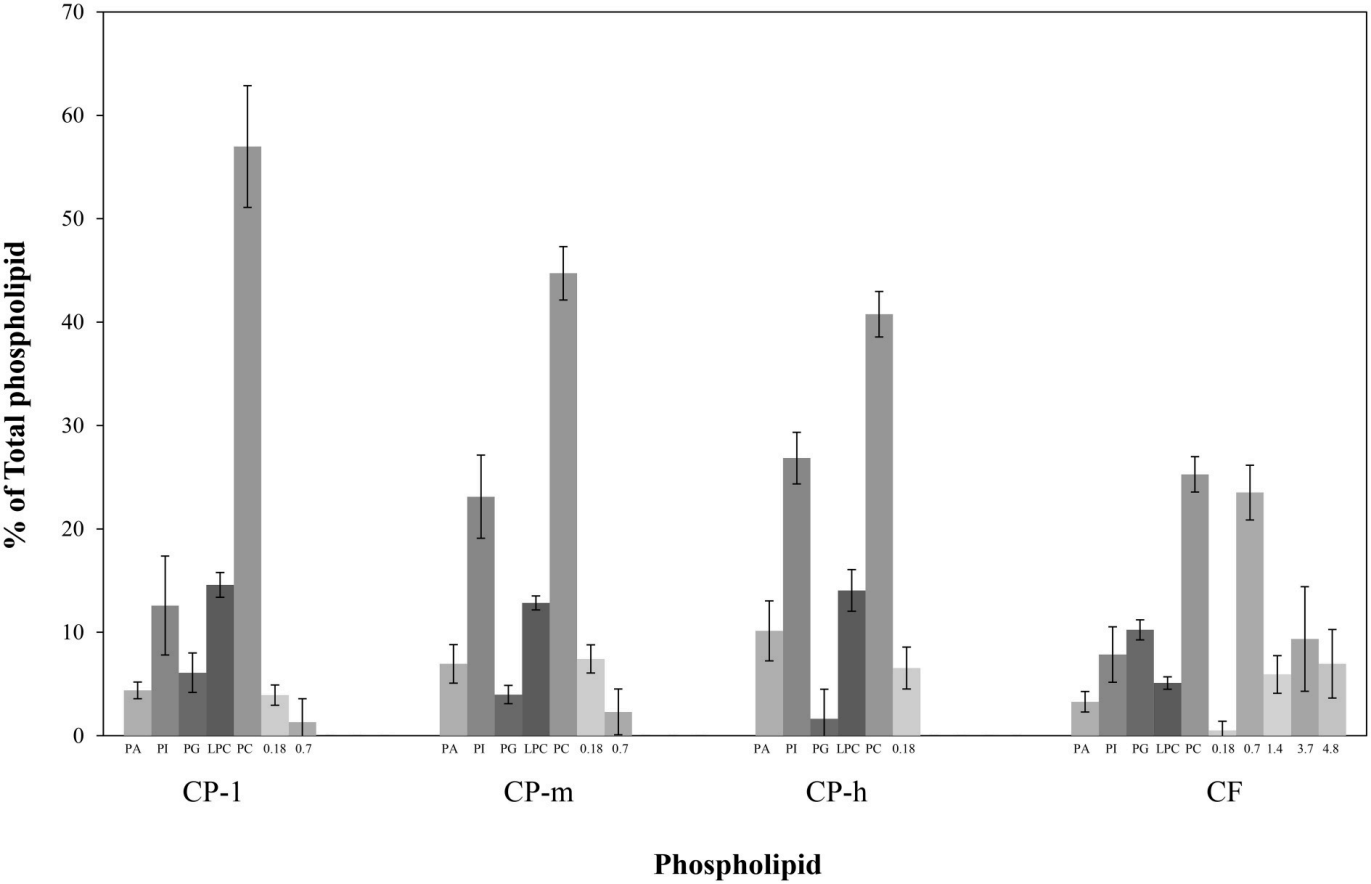
**Profiling of phospholipid fraction of four cocoa materials using ^31^P NMR**. PA, Phosphatidic acid; PG, Phosphatidylglycerol; PI, Phosphatidylinositol; LPC, *Lyso*-phosphatidylcholine; and PC, Phosphatidylcholine. The contribution of the total phospholipid which was not identified is presented by the chemical shift (ppm). CP-l, m and h indicate the low, medium and high lipid and triglyceride fraction of the original cocoa powders respectively. Error bars denote standard deviation.

The relative proportions of the phospholipids were determined by integrating the resonances of the different phosphorus environments. This indicated the composition of the phospholipid fraction of each cocoa material (Figure 3). Phosphatidylcholine (PC) dominated in each type of cocoa material. Not all of the signals could be identified, however, unidentified signals contributed less than 10% of the total phospholipid for the cocoa powders. ^31^P NMR spectra of all samples showed additional peaks at chemical shifts of 0.18 and 0.7 ppm. The peak at 0.75 ppm may be evidence for the presence of cardiolipin (Culeddu et al., 1998), which was present in a much higher concentration for CF (24%). The unknown signals (0.18, 1.4, 3.7, and 4.8 ppm) may be ascribed to small molecules, e.g., glycerol phosphate. Inorganic phosphates such as sodium phosphate, and pyrophosphates such as ADP can be ruled out as they do not dissolve in the solvent system used to disperse lipids samples for NMR spectroscopy. Full structural determination is required for unambiguous identification of these phosphorylated species.

### Characterization of the emulsifying ability of delipidified cocoa material

**Figure 4:**
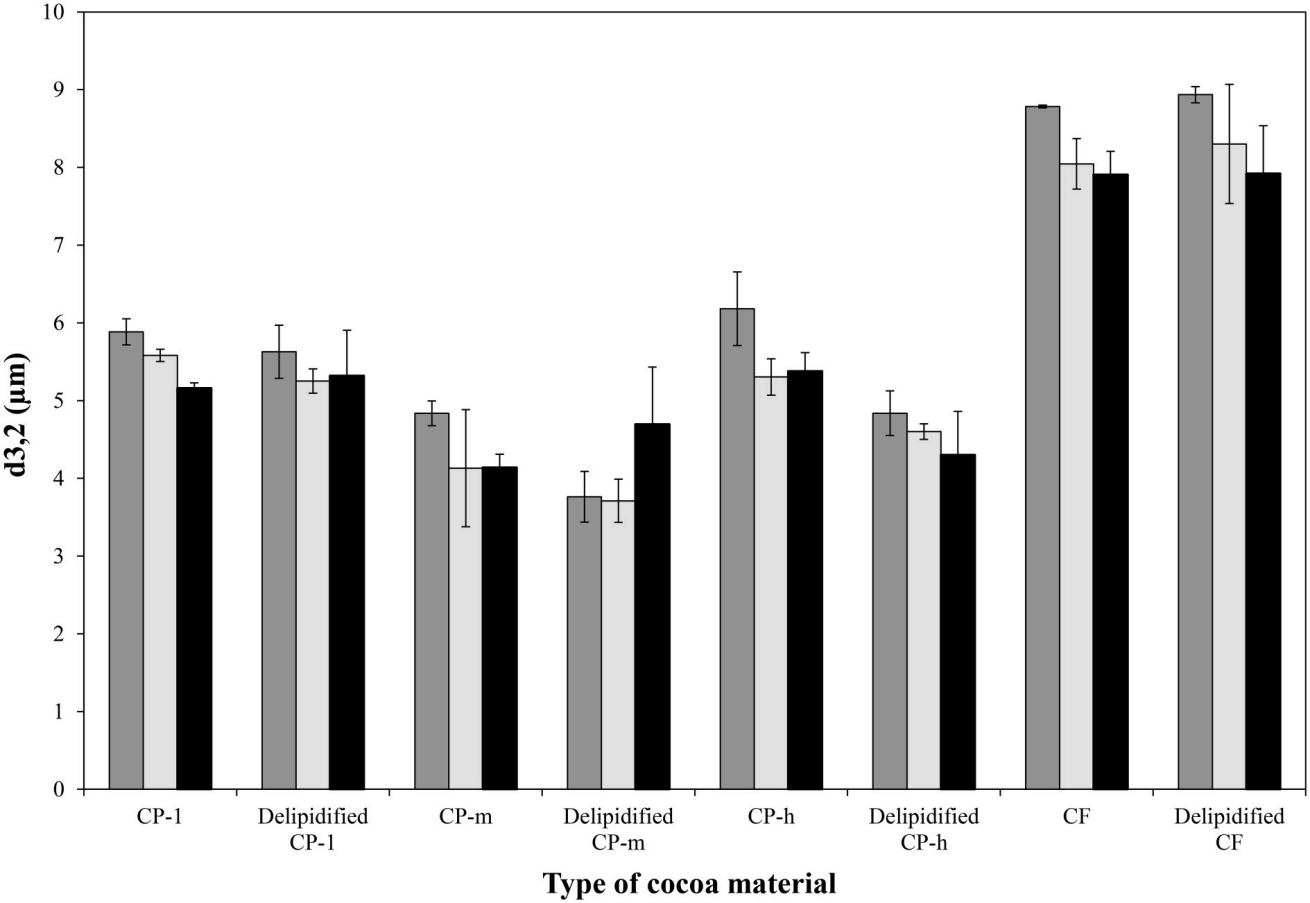
**Droplet diameter of emulsions stabilized with untreated and delipidified cocoa material measured after 1 day (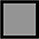), 53 days (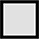) and 100 days (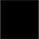) of storage at 20°C**. CP-l, m and h indicate the low, medium, and high lipid and triglyceride fraction of the original cocoa powders respectively. The error bars denote standard deviation.

All types of delipidified cocoa material stabilized o/w emulsions. Figure 4 shows that there was no significant increase in droplet size of the emulsions stabilized with untreated or delipidified cocoa material measured over 100 days of storage at 20°C (*p* < 0.05). In addition there was no evidence of a coalesced oil layer after 2 years of storage at 20°C. This indicates that the removal of lipid did not affect the coalescence stability of cocoa stabilized o/w emulsions.

While none of the emulsions showed coalescence, in the case of the cocoa powders with medium and high lipid content (CP-m and CP-h, respectively) the removal of the lipid fraction had a significant impact on the mean droplet diameter of the emulsions. In both cases it was significantly lower following lipid extraction compared to the mean diameter of the emulsions prepared with the original cocoa material (*p* = 0.01 and *p* = 0.02, respectively). As emulsion droplet size has a linear relationship with the size of the particles stabilizing the interface (Binks and Lumsdon, 2001), the particle size of the cocoa material was evaluated. Particle size measurement of the cocoa material, shown in Table 2, confirms that lipid extraction caused a significant reduction in particle size of cocoa materials CP-m and CP-h. There was no significant change in particle size of CP-1 or CF.

**Table 2:** **Particle size (d_3, 2_) of aqueous dispersions of untreated and delipidified cocoa material**.

**Type of cocoa material**	**Particle size (d**_**3, 2**_**) (μm)**	
	**Untreated**	**Delipidified**
CP-l	9.58 ± 0.24	9.90 ± 0.37
CP-m	10.58 ± 0.13	8.02 ± 0.76^*^
CP-h	13.66 ± 1.03	8.27 ± 0.31^*^
CF	16.87 ± 0.57	18.28 ± 1.69

CP-l, m and h indicates the low, medium and high lipid content of the original cocoa powders respectively. The effect of lipid extraction on particle size was statistically tested per cocoa material type. The presence of an asterisk (

* *) indicates the particle size of the delipidified material was significantly different (p < 0.05) to the untreated material. Differences between the types of cocoa material were not evaluated*.

### Surface tension of delipidified cocoa material

The surface tension of the aqueous dispersions of the four cocoa materials was measured to assess whether the phospholipid fraction contributes to the interfacial activity of cocoa as phospholipids are known to facilitate emulsion formation by reducing the interfacial tension. The surface tension of the aqueous dispersion of the cocoa material analyzed after 600 s are shown in Table 3.

**Table 3:** **Surface tension of aqueous dispersions of untreated and delipidified cocoa material**.

**Type of cocoa material**	**Surface tension (mN.m^−1^)**
CP-l	47.82 ± 0.13^c^
delipidified CP-l	48.84 ± 0.00^c^
CP-m	44.43 ± 0.73^b^
delipidified CP-m	50.35 ± 0.66^c^
CP-h	40.78 ± 0.59^a^
delipidified CP-h	50.05 ± 0.91^c^
CF	37.99 ± 0.87^a^
delipidified CF	48.34 ± 2.59^c^

*CP-l, m and h indicate the low, medium and high lipid content of the original cocoa powders respectively. Mean surface tension values after 600 s of measurement at 20°C are presented. The different letters represent a significant difference (p < 0.05)*.

These data indicate that lipid extraction significantly decreased the surface tension of the cocoa material except in the case of the cocoa powder with the smallest proportion of lipid (CP-l). Surface tension measurements of the aqueous dispersions of the cocoa material indicated that the phospholipids contribute to the surface activity of the dispersions as removal of the lipid fraction decreased the surface tension.

## Discussion

In this study the contribution of the endogenous lipid of cocoa material was evaluated with respect to the emulsifying ability of cocoa. The phospholipid fraction, quantified by mass after extraction using organic solvents, was consistent with supplier specifications and removal of the entire lipid fraction from the cocoa material. Notably, the phospholipid fraction [0.9–2.4% (*w/w*)] was higher than the 0.3–0.4% (*w/w*) previously reported for cocoa beans (Knapp, 1937; Parsons et al., 1969). However, the techniques used previously were based on chromatographic rather than spectroscopic methods and thus did not produce structural data to support identification of the compounds present. The comparative advantages of the ^31^P NMR spectroscopy is that it is a high resolution, quantitative technique that gives structural data. Furthermore, the method used to isolate the lipid fraction in the current study was designed to isolate lipids with a variety of head groups. Earlier methods were less general (Rydhag and Wilton, 1981; Furse et al., 2015a). This may be why previously unreported phosphorylated species (unknown species, Figure 3) were observed.

^31^P NMR spectroscopy of the cocoa indicated the presence of phospholipids in all types of cocoa material tested with five well-known phospholipids (PA, PG, PI, LPC, and PC) identified in all samples. The proportion of each of phospholipid varied between the types of cocoa material although PC was the major phospholipid [25–57% (*w/w*)] in all samples. The same high concentration of PC was previously reported for cocoa beans where PC was found to contribute 36–40% of the total phospholipid (Parsons et al., 1969). The presence of PC was of particular interest as PCs are a major component of lecithin; a commonly used emulsifier (Whittinghill et al., 2000; Pichot et al., 2013).

The contribution of the (whole) lipid fraction to the emulsifying ability of the cocoa material was quantified by a comparison of the emulsions generated by cocoa before and after extraction of the lipid fractions. All four cocoa materials were found to be able to stabilize an o/w emulsion after delipification. There was no significant increase in emulsion droplet diameter over a period of 100 days which indicates that emulsions were stable to droplet coalescence. Lipid extraction did affect the droplet size of two of the emulsion samples prepared from cocoa materials of originally medium and high lipid content. This may be ascribed to the change in the size distribution of the cocoa material to smaller diameters following lipid extraction, as shown in Table 2, as smaller particle diameters are known to enable stabilization of smaller droplets (Binks and Lumsdon, 2001; Luo et al., 2011). The shift in size distribution of the particles may be due to removal of surface lipid that promotes particle aggregation.

The role of the lipid fraction on the interfacial properties of commercially available cocoa material was quantified by analysis of the surface tension of aqueous dispersions of both untreated and delipidified cocoa material. We found that the lipid present in cocoa contributed to the surface activity of the dispersions. This is consistent with the established behavior of phospholipid as a surfactant. However, extracting the lipids decreased the surface tension to values comparable to the cocoa powder with the originally smallest lipid fraction, (CP-l), which was still capable of driving the formation of an emulsion. The aqueous dispersions of delipidified cocoa material were still surface active which may explain why emulsions could be formed by the delipidified cocoa. However, the stability of particle stabilized emulsions is not reliant upon particle adsorption at the interface changing the interfacial tension as dispersions with known emulsifying ability have been shown not to decrease interfacial tension (Tzoumaki et al., 2011; Rana et al., 2012).

## Conclusion

The evidence from this study indicates that several phospholipids found in lecithin and other known surfactant mixtures are present in cocoa material. Crucially, there is evidence that the formation of o/w emulsions based on cocoa material is not only driven by these molecules but also by other biomolecules that do not dissolve in organic solvents or water. This observation raises a number of questions. Naturally, what this component is and how it works are important but also where else such components are found and how they may be developed for commercial use. Further research is required to characterize this emulsifier properly. Cocoa is an heterogeneous material containing lipids, polyphenol, proteins, starch and lignin, all of which have known emulsifying ability. We suggest that the next step in elucidating the behavior of these systems is to evaluate role of these components on the emulsifying ability of the cocoa material. The results of such a study would shape efforts in identifying other naturally occurring material with emulsifying ability as well as preparing emulsifying material from natural material.

## Author contributions

BW wrote the original grant proposal. JG and BW conceived the research project and designed surface tension experiments. JG carried out all surface tension experiments and isolated lipid samples. SF supervised lipid isolation, ran NMR spectroscopy experiments and analyzed NMR data. JG drafted the manuscript; all authors were involved in revising it; BW and SF supervised its preparation. All authors (JG, BW, SF) have approved and are accountable for the final version of the manuscript submitted.

### Conflict of interest statement

The authors declare that the research was conducted in the absence of any commercial or financial relationships that could be construed as a potential conflict of interest.
